# Sugar metabolism and the plant target of rapamycin kinase: a sweet operaTOR?

**DOI:** 10.3389/fpls.2013.00093

**Published:** 2013-04-15

**Authors:** Thomas Dobrenel, Chloé Marchive, Marianne Azzopardi, Gilles Clément, Manon Moreau, Rodnay Sormani, Christophe Robaglia, Christian Meyer

**Affiliations:** ^1^Institut Jean-Pierre Bourgin, UMR 1318 INRA AgroParisTech, Saclay Plant SciencesVersailles, France; ^2^Laboratoire de Génétique et Biophysique des Plantes, UMR7265, DSV, IBEB, SBVME, CEA, CNRS, Faculté des Sciences de Luminy, Aix Marseille UniversitéMarseille, France

**Keywords:** target of rapamycin, starch, raffinose, *myo*-inositol-1-phosphate synthase, TOR serine-threonine kinases

## Abstract

In eukaryotes, the ubiquitous TOR (target of rapamycin) kinase complexes have emerged as central regulators of cell growth and metabolism. The plant TOR complex 1 (TORC1), that contains evolutionary conserved protein partners, has been shown to be implicated in various aspects of C metabolism. Indeed *Arabidopsis* lines affected in the expression of TORC1 components show profound perturbations in the metabolism of several sugars, including sucrose, starch, and raffinose. Metabolite profiling experiments coupled to transcriptomic analyses of lines affected in TORC1 expression also reveal a wider deregulation of primary metabolism. Moreover recent data suggest that the kinase activity of TORC1, which controls biological outputs like mRNA translation or autophagy, is directly regulated by soluble sugars.

## INTRODUCTION

The adjustment of primary metabolism to environmental conditions and to the availability of energy and nutrients is of primary importance to maintain cell homeostasis. Plants, like other eukaryotic organisms, have evolved to make an optimal use of nutrients and to adapt to nutritional deficiencies. This implies that plants have the ability to monitor the amount of available nutrients and energy and to adapt their transcriptional, translational, and metabolic responses to this information. In animals, in which cells are continuously maintained in a rather buffered and uniform supply of nutrients, this regulation of metabolic activity and cell growth at the cellular level is mainly driven by growth factors and hormones. For plants, nutrients provide not only the food for growth but also the signals for growth. Indeed nutrients serve both as the resources by which the cell increases mass and generates energy and as the signals controlling the metabolic and developmental programs which optimize survival under particular nutritional states.

Furthermore, plants experience rapid, sudden, and often long changes from optimal growth conditions and they must be able to both monitor precisely these changes and to trigger counter-measures ensuring survival and adaptation while maintaining growth and biomass production. In plants, like in other eukaryotes, the signaling pathway involving the TOR (target of rapamycin) protein kinase has emerged as an evolutionary conserved and critical link between external cues and metabolic and growth adaptations (see Wullschleger et al., 2006; Ma and Blenis, 2009; Loewith and Hall, 2011; Laplante and Sabatini, 2012; Cornu et al., 2013 for general reviews and Dobrenel et al., 2011; John et al., 2011; Robaglia et al., 2012 for reviews on the plant TOR signaling pathway).

Target of rapamycin was identified 20 years ago in yeast in a screen for mutations conferring resistance to rapamycin, an antibiotic that stops growth and induces a shift to the G0 quiescent stage (Heitman et al., 1991). It was later shown that rapamycin inhibits TOR by triggering the formation of an artificial complex between the TOR FRB (FKBP12-rapamycin binding) domain and the small FKBP12 protein (Wullschleger et al., 2006). Rapamycin treatment inhibits some of the TOR-linked activities and results, in yeast and animal cells, in the accumulation of the storage compound glycogen, in translation decrease and in the induction of autophagy (Schmelzle et al., 2004; Rohde et al., 2008; Broach, 2012; Cornu et al., 2013). These changes also occur in nutrient-starved cells (Rohde et al., 2008; Broach, 2012), which suggests that TOR is one of the main components of the transduction chain linking nutrient signaling to cellular adaptations. Indeed a wealth of studies, both in yeast and in animals, have clearly established that the TOR kinase is activated by external signals like the availability of amino acids or the presence of hormones, and then controls a myriad of biological outputs including transcription of RNA, translation, ribosome biogenesis, translocation of regulatory proteins, autophagy, and storage of reserve compounds (see above reviews). This review will mainly focus on the cross-talk between the conserved plant TOR kinase signaling pathway and C metabolism with a particular emphasis on C storage compounds.

## THE TOR KINASE

The large TOR kinase associates in high molecular mass complexes with other conserved protein partners (Wullschleger et al., 2006; Loewith and Hall, 2011; Wang and Proud, 2011). In yeast and animals, the TOR kinase functions in two distinct multiprotein complexes named TOR complex 1 (TORC1) and TOR complex 2 (TORC2). The rapamycin-sensitive TORC1 contains three major proteins (TOR, KOG1/RAPTOR, and LST8/GbetaL), which are also found in plants (Menand et al., 2002; Anderson et al., 2005; Deprost et al., 2005; Mahfouz et al., 2006; Moreau et al., 2012) and is thought to mainly regulate metabolism, mRNA translation, and autophagy (Wang and Proud, 2011). The TOR/RAPTOR and TOR/LST8 interactions have also been established in plants (Mahfouz et al., 2006; Díaz-Troya et al., 2008; Moreau et al., 2012). The TORC2 complex contains LST8/GbetaL with specific proteins like AVO3/RICTOR and AVO1/SIN1 (Wullschleger et al., 2006). The existence of the TORC2 complex in plants has not been proven so far but it may represent a more recent addition to the TOR signaling pathway.

It was previously thought that rapamycin, even at important doses, does not affect plant growth. Indeed plant FKBP12 proteins carry mutations that would preclude the formation of a TOR–rapamycin–FKBP12 ternary complex (Xu et al., 1998; Menand et al., 2002; Mahfouz et al., 2006; Sormani et al., 2007). Accordingly no interactions were detected between *Arabidopsis* TOR and FKBP12 proteins using two-hybrid techniques (Mahfouz et al., 2006; Sormani et al., 2007). However, the *Arabidopsis* TOR FRB domain could bind the yeast (Sormani et al., 2007) or human (Mahfouz et al., 2006) FKBP12 proteins in the presence of rapamycin. This opened the possibility of increasing plant sensitivity toward rapamycin by expressing the yeast FKBP12 protein (Sormani et al., 2007; Leiber et al., 2010; Ren et al., 2012). Conversely, the unicellular green alga *Chlamydomonas* is sensitive to moderate levels of rapamycin (100–500 nM), a concentration range similar to the one necessary to inhibit yeast growth (Heitman et al., 1991; Crespo et al., 2005). This can be explained by the fact that the algal FKBP12 protein is closer to human or yeast homologs and the residues critical for binding rapamycin are conserved only in *Chlamydomonas*. Nevertheless, it was recently shown that rapamycin, when added repeatedly at high concentrations (1–10 μM) to liquid cultures of *Arabidopsis*, could affect plant growth and development (Xiong and Sheen, 2012).

This varying and reduced susceptibility of plants to rapamycin has clearly delayed the development of molecular studies on the plant TOR signaling pathway. Moreover the disruption of the AtTOR gene by T-DNA insertions was shown to be embryo lethal (Menand et al., 2002; Ren et al., 2011), which precluded the use of these mutants to further study the role of TOR in plants. To circumvent these difficulties we have produced constitutive and ethanol-inducible RNAi lines which allow a stable or conditional silencing of the AtTOR gene (Deprost et al., 2007). This study and other reports using estradiol-inducible artificial microRNA (amiRNA) showed that, when the expression of AtTOR was silenced, plant growth was arrested and several metabolites accumulated, including starch, triacylglycerides (TAGs), and amino acids (Deprost et al., 2007; Dobrenel et al., 2011; Xiong and Sheen, 2012; Caldana et al., 2013). This was accompanied by vast modifications in the plant transcriptome. *Arabidopsis* plants silenced for the AtTOR expression also displayed a significant reduction in polysome abundance (Deprost et al., 2007), in the phosphorylation of the ribosomal S6 kinase (S6K, Schepetilnikov et al., 2011; Xiong and Sheen, 2012) and were presenting signs of constitutive autophagy (Liu and Bassham, 2010). These results suggest that the main biological targets of the yeast and animal TORC1 complex, namely, S6K, mRNA translation, and autophagy are conserved during evolution. All these *Arabidopsis* lines provided invaluable tools to start deciphering the metabolic consequences of the inhibition of TOR activity in time-course experiments. It should be stressed that TOR inhibition by RNAi is likely to reveal a larger spectrum of phenotypes than rapamycin since this drug is known to inhibit only a subset of TORC1 activities, and not the TORC2 complex (Feldman et al., 2009; Guertin and Sabatini, 2009; Thoreen et al., 2009). Accordingly recent data suggest that knocking out TOR activity by silencing has more profound consequences than partly inhibiting the TORC1 complex with rapamycin (Ren et al., 2012).

## REGULATION OF THE TORC1 COMPLEX BY SUGARS

In yeast it has been shown that carbon or nitrogen starvation inhibits TORC1 activity and that rapamycin action mimics the effects of nutrient removal by, for example, inducing autophagy or the expression of genes involved in the utilization of alternative source of nutrients (Rohde et al., 2008; Broach, 2012). It was for a long time unclear how nutrients regulated TORC1 activity, but recent reports nicely demonstrated that the vacuolar H^+^-ATPase (v-ATPase) activates the TORC1 complex by recruiting it to the surface of yeast vacuoles or animal lysosomes in the presence of amino acids (Binda et al., 2009; Zoncu et al., 2011). This recruitment of TOR and the subsequent increase in TORC1 activity are mediated by the Rheb and Rag GTPase complexes (Cornu et al., 2013). Very recently it was found that glucose also induces TOR activity by regulating the binding of the v-ATPase to Rag GTPases, thus suggesting a shared regulatory mechanism between sugars and amino acids (Efeyan et al., 2013). Moreover the glycolytic enzyme glyceraldehyde-3-phosphate dehydrogenase (GAPDH) binds Rheb in low-glucose conditions and inhibits mTORC1 (mammalian target of rapamycin complex 1) signaling (Lee et al., 2009). Interestingly, in plants, the v-ATPase has also important roles in nutrient storage and signaling (Schumacher and Krebs, 2010). Similarly glucose, an important plant regulatory molecule, has been shown to be linked to TOR activation in *Arabidopsis* (Xiong and Sheen, 2012). The class III/Vps34 PI3K (phosphoinositide 3-kinase) has also been involved in nutrient activation of TORC1 through the production of PI3P (Gulati et al., 2008). Since this kinase is well-conserved in plants and affects the TOR signaling pathway (Turck et al., 2004), it would be quite interesting to evaluate its contribution to the nutrient regulation of TORC1.

## ROLE OF THE PLANT TORC1 COMPLEX IN STARCH AND RAFFINOSE ACCUMULATION

**FIGURE 1:**
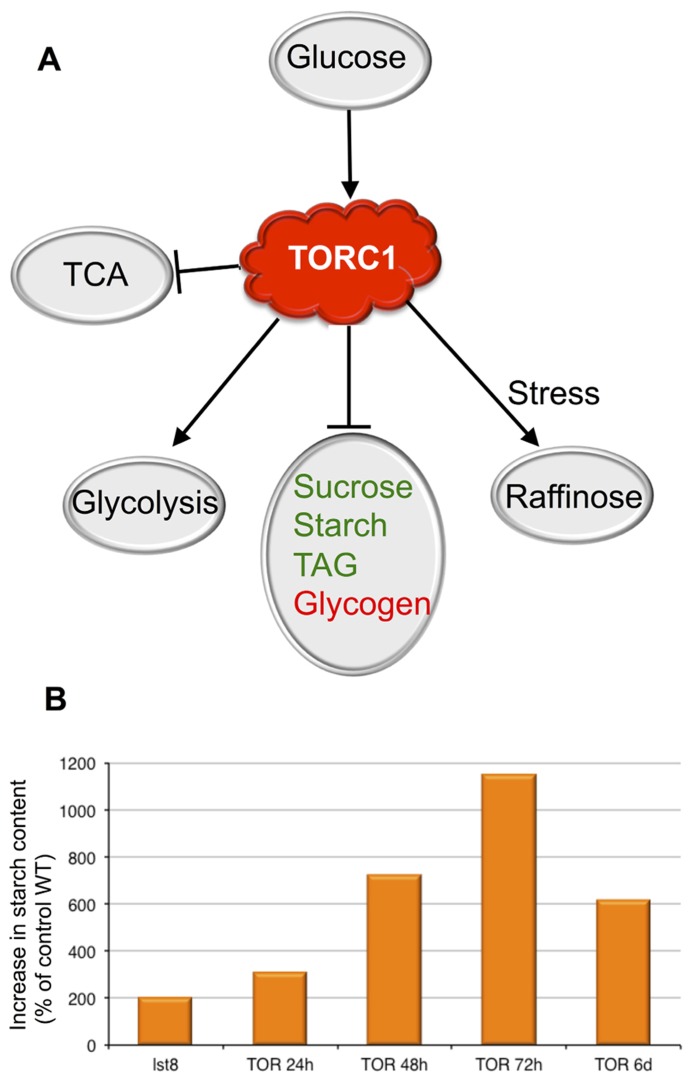
**The roles of theTOR kinase in the regulation of C metabolism.** **(A)** Summary of the cross-talk between the TORC1 signaling pathway and the sugar metabolism. Glucose seems to induce TOR activity, which in turn activates glycolysis and raffinose synthesis in response to stresses. Conversely TOR represses the synthesis of reserve molecules. Animal reserve compounds are in red, plant ones are in green. Activation is shown by arrows. **(B)** Accumulation of starch following inactivation of the TORC1 complex in percentage of the control wild-type (WT). The effect of the inactivation of the TORC1 component LST8 on starch accumulation was investigated in insertion mutants. Results from the analysis of *lst8* mutants are from Moreau et al. (2012) and are compared to the corresponding control WT (Col8). A time-course experiment shows the accumulation of starch following inactivation of TOR by ethanol-inducible RNAi (24–72 hours (h) after ethanol induction; Deprost et al., 2007). The 6 days (6d) point is from Caldana et al. (2013) using estradiol-inducible amiRNA lines.

Inhibition of TOR activity results in C storage through glycogen accumulation in animal muscles and yeast (Schmelzle et al., 2004; Cornu et al., 2013). Conversely TOR inhibition in the liver decreases the level of stored glycogen and the animals become hyperglycemic, a situation also found in type 2 diabetes. This suggests a prominent role of TOR in maintaining animal glucose homeostasis (Cornu et al., 2013). In yeast TORC1 inhibition by rapamycin triggers a switch from fermentation to respiration by reducing the expression of genes encoding glycolytic enzymes and increasing the expression of genes encoding tri carboxylic acid (TCA) enzymes (**Figure 1A**; De Virgilio and Loewith, 2006). The TORC1 signaling pathway is evidently important for cell metabolism and proliferation, therefore its perturbation is implicated in many human diseases (Cornu et al., 2013). Indeed activation of the TOR kinase is frequently encountered in human cancers and has been found to promote the flux of C through glycolysis by up-regulating genes involved in glucose uptake and glycolysis. TOR activation also stimulates lipogenesis and the pentose phosphate pathway (Düvel et al., 2010; Yecies and Manning, 2011).

In plants most of the stored C is found in starch. Several studies have established that starch is mainly synthesized during the day within the chloroplasts and degraded at night to provide cells with C metabolites and energy (for an excellent and recent review see Stitt and Zeeman, 2012). When limited in nitrogen, *Chlamydomonas* cells accumulate starch (Ball et al., 2011) and TAG (Siaut et al., 2011). Similarly, plants affected in nitrate assimilation accumulate starch at high levels (Saux et al., 1987). These results point clearly to a control of starch synthesis by the availability of nitrogen. Indeed in conditions where nitrogen is limiting, and growth is therefore reduced, most of the available C seems to be redirected toward the accumulation of reserves rather than toward other sinks like the synthesis of cell wall or the production of energy. Consistently, metabolite profiling of *Arabidopsis* accessions has shown that starch and sucrose contents are negatively correlated with rosette growth in *Arabidopsis* (Sulpice et al., 2009, 2010).

Conditional silencing of the *Arabidopsis* TOR gene led to a decrease in photosynthesis with a yellowing of the leaves linked to chlorophyll breakdown (Deprost et al., 2007). These symptoms of early senescence where accompanied by an accumulation of high amounts of soluble sugars, amino acids, and starch (**Figure 1B**). This suggests that TOR activity is needed to restrain senescence and could thus be involved in the regulation of life span in *Arabidopsis* (Deprost et al., 2007; Ren et al., 2012). A recent study also showed that TOR inhibition in *Arabidopsis* by inducible amiRNA results in high levels of starch accumulation (**Figure 1B**) together with increased levels of TAG (Caldana et al., 2013). A concomitant increase in TCA cycle intermediates was also detected after TOR inhibition by either amiRNA (Caldana et al., 2013), the treatment by rapamycin of *Arabidopsis* lines expressing a FKBP12 protein (Ren et al., 2012) or in *lst8* mutants (Moreau et al., 2012). The same perturbation in the TCA cycle was observed in yeast treated with rapamycin (De Virgilio and Loewith, 2006).

**FIGURE 2:**
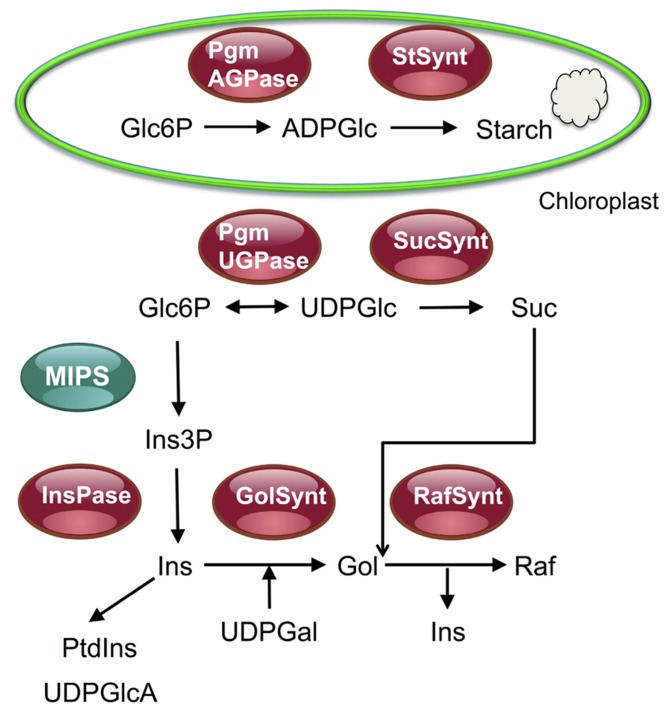
**Overview of the raffinose synthesis pathway and of its relationship with the sucrose and starch synthesis pathways.** This figure shows that glucose 6-phosphate (Glc6P) can be used either for the synthesis of starch in chloroplasts or for the synthesis of sucrose or *myo*-inositol and raffinose in the cytosol. After TOR inactivation, or mutations in the TORC1 components RAPTOR and LST8, an accumulation of starch and sucrose was observed together with a decrease in the synthesis of raffinose in stress conditions. This suggests TOR inactivation triggers a redirection of C fluxes toward starch and sucrose for, respectively, storage and export. Pgm, phosphoglucomutase; StSynt, starch synthase; AGPase, ADPglucose pyrophosphorylase; UGPase, UDPglucose pyrophosphorylase; SucSynt, sucrose synthase; MIPS, *myo*-inositol 3-phosphate synthase; Ins(3P), *myo*-inositol (3 phosphate); InsPase, inositol monophosphate phosphatase; PtdIns, phosphatidylinositol; UDPGlcA, UDP-glucuronic acid; Gol(Synt), galactinol (synthase); Raf(Synt), raffinose (synthase).

In the study by Sulpice et al. (2009) described above, it was found that rosette biomass negatively correlated with the amount of starch but a strong positive correlation was detected with the expression of *myo*-inositol-1-phosphate synthase 1 (MIPS1/At4g39800). MIPS is conserved in all eukaryotes and catalyzes the first committed step in the synthesis of *myo*-inositol, a central C-metabolite that serves in the synthesis of the signaling lipids (phosphatidyl)inositol-phosphate (PIP), of cell wall precursors like UDP-glucuronate and of the raffinose family oligosaccharides (RFOs, **Figure 2**; for a review see Valluru and Van den Ende, 2011). The levels of raffinose, of its precursor galactinol and of *myo*-inositol, which serves as a cofactor in this biosynthetic pathway, are often strongly correlated (**Figure 2**; G. Clément, personal communication; Sulpice et al., 2010). A survey of metabolite profiling experiments shows that raffinose accumulates in stress situations like high light, nitrogen starvation, or high salt (G. Clément, personal communication and unpublished data).

A common trend that emerges from the analysis of stressed *Arabidopsis* plants affected in the activity of the TORC1 complex is the decrease in raffinose and galactinol accumulation. Ren et al. (2012) observed lower levels of raffinose and *myo*-inositol in rapamycin-treated plants expressing a yeast FKBP12 protein in normal growth conditions. However, a decrease in the accumulation of these sugars and of galactinol was only evident in stressed TOR RNAi lines compared to control wild-type plants or when *lst8* mutants were exposed to long days (Moreau et al., 2012). Similarly, stressed *raptor* mutants fail to accumulate raffinose and galactinol (Szambien et al., submitted). Galactinol is synthesized from UDP-Gal and *myo*-inositol in the cytosol (**Figure 2**). Galactinol then serves for raffinose production using sucrose as a substrate, with a release of *myo*-inositol (**Figure 2**; Nishizawa et al., 2008; Valluru and Van den Ende, 2011). Raffinose and galactinol usually accumulate in seeds and in response to many stresses and could function as storage C molecules (Taji et al., 2002; Peters et al., 2010; Valluru and Van den Ende, 2011). It has been proposed that one of the main role of these polysaccharides is to scavenge reactive oxygen species in the cytosol and in the chloroplast (Keunen et al., 2013). Moreover RFOs represent significant sinks for glucose- and sucrose-derived C since they can accumulate at rather high concentrations.

Like *lst8* mutants (Moreau et al., 2012), plants affected in the MIPS1 gene expression were described as lacking galactinol accumulation and being sensitive to long days (Meng et al., 2009). Accordingly the transcriptome variations due to either *mips1* or *lst8* mutations shared some striking similarities. Indeed, nearly 70% of the genes differentially expressed in the *lst8* mutant when shifted to long days were also found to be either down- or up-regulated in the *mips1* mutant (Moreau et al., 2012). This indicates that a large proportion of the impact of *lst8* mutations, which probably result in a decreased TORC1 activity, on the adaptation to long days can be explained by a default in MIPS1 activity and thus by a decrease in *myo*-inositol production. This default in raffinose and galactinol production after extension of the light period could explain the arrest of growth and the severe phenotype of both *lst8* and *mips1* mutants following a shift to long day conditions.

Furthermore MIPS and galactinol synthases were found to be significantly repressed in TOR RNAi lines or in the *lst8* mutants grown in long days. These results are in agreement with the observed lack of galactinol and raffinose accumulation in plants where the TORC1 activity is reduced (Moreau et al., 2012; Ren et al., 2012). The MIPS genes could therefore serve as a hub for adjusting the plant metabolism to changes in environmental conditions. The inducible overexpression of the bZIP11 transcription factor, which is normally up-regulated by the SnRK1 kinase, results in an augmented level of raffinose (Ma et al., 2011). This is consistent with the fact that raffinose is also accumulated in response to multiple stresses (Valluru and Van den Ende, 2011). Indeed this could possibly be the result of the activation of the SnRK1 kinase in stress conditions (Robaglia et al., 2012). Nevertheless it is surprising, given the expected opposite role of the TOR and SnRK1 kinases (Robaglia et al., 2012), that TOR also seems to be required for raffinose production. One explanation could be that TORC1 activity is also needed for bZIP11 expression and it would be interesting to determine if the bZIP11-induced accumulation of raffinose is TOR-dependent.

## CONCLUSION

It is now clear that inhibiting the TORC1 activity results in starch and TAG accumulation (Dobrenel et al., 2011; Caldana et al., 2013), a decrease in biomass production but also a decrease in protein concentration and mRNA translation (Deprost et al., 2007; Sormani et al., 2007; Ren et al., 2012; Xiong and Sheen, 2012; Caldana et al., 2013). It thus appears that the TOR signaling pathway may contribute to the close link between starch, protein, and biomass observed in plants (Sulpice et al., 2009, 2010). The signals triggering starch accumulation and re-routing of C fluxes in response to TORC1 inactivation remain to be determined, but it is striking that the accumulation of starch observed in TORC1-deficient *Arabidopsis* plants is accompanied by a decrease in raffinose production, both being dependent on the supply of glucose-6P (**Figure 2**). The TORC1 activity is probably also required for plant adaptation to stresses by stimulating synthesis of *myo*-inositol and RFOs. Whether they serve as C storage molecules or for the scavenging of reactive oxygen species remains to be determined more clearly.

Since the inactivation of TOR results, as in other eukaryotes, in the accumulation of reserve molecules in plants (TAG and starch), it can be anticipated that the regulation of TOR activity in developing seeds may also be of importance for the synthesis of seed storage compounds. Moreover, using TOR inactivation to redirect C fluxes toward reserves compounds like starch or TAG, which are easier to process than lignocellulosic molecules, could foster the use of plants for the production of biofuels and other bio-based components.

## Conflict of Interest Statement

The authors declare that the research was conducted in the absence of any commercial or financial relationships that could be construed as a potential conflict of interest.
